# *Bifidobacterium breve* and *Lactobacillus rhamnosus* treatment is as effective as budesonide at reducing inflammation in a murine model for chronic asthma

**DOI:** 10.1186/1465-9921-15-46

**Published:** 2014-04-16

**Authors:** Seil Sagar, Mary E Morgan, Si Chen, Arjan P Vos, Johan Garssen, Jeroen van Bergenhenegouwen, Louis Boon, Niki A Georgiou, Aletta D Kraneveld, Gert Folkerts

**Affiliations:** 1 Division of Pharmacology, Utrecht Institute for Pharmaceutical Sciences, Faculty of Science, Utrecht University, PO box 80082, 3508 TB Utrecht, The Netherlands; 2 Danone Research, Centre for Specialised Nutrition, Wageningen, The Netherlands; 3 Bioceros B.V., Utrecht, The Netherlands

**Keywords:** Allergic asthma, Beneficial bacteria, Glucocorticoids, Regulatory T cell

## Abstract

**Background:**

Asthma is estimated to affect as many as 300 million people worldwide and its incidence and prevalence are rapidly increasing throughout the world, especially in children and within developing countries. Recently, there has been a growing interest in the use of potentially beneficial bacteria for allergic diseases. This study is aimed at exploring the therapeutic effects of long-term treatment with two different beneficial bacterial strains (*Bifidobacterium breve* M-16 V and *Lactobacillus rhamnosus* NutRes1) and a glucocorticoid (budesonide), as a reference treatment, on inflammatory response in a murine model for chronic allergic asthma.

**Methods:**

To mimic the chronic disease in asthmatic patients, we used the murine ovalbumin-induced asthma model combined with prolonged allergen exposure. Airway function; pulmonary airway inflammation; airway remodelling, mRNA expression of pattern recognition receptors, Th-specific cytokines and transcription factors in lung tissue; mast cell degranulation; *in vitro* T cell activation; and expression of Foxp3 in blood Th cells were examined.

**Results:**

*Lactobacillus rhamnosus* reduced lung resistance to a similar extent as budesonide treatment in chronically asthmatic mice. Pulmonary airway inflammation, mast cell degranulation, T cell activation and airway remodelling were suppressed by all treatments. Beneficial bacteria and budesonide differentially modulated the expression of toll-like receptors (TLRs), nod-like receptors (NLRs), cytokines and T cell transcription factors. *Bifidobacterium breve* induced regulatory T cell responses in the airways by increasing *Il10* and *Foxp3* transcription in lung tissue as well as systemic by augmenting the mean fluorescence intensity of Foxp3 in blood CD4+ T cells.

**Conclusion:**

These findings show that *Bifidobacterium breve* M-16 V *and Lactobacillus rhamnosus* NutRes1 have strong anti-inflammatory properties that are comparable to budesonide and therefore may be beneficial in the treatment of chronic asthma.

## Background

Allergic asthma is a T helper type-2 (Th2) cell-mediated chronic inflammatory disorder of the airways with rapidly increasing incidence and prevalence throughout the world, especially in children and within developing countries [1]. It is characterised by airway inflammation and hyper-responsiveness (AHR) [2]. Structural changes in the airway walls of asthmatic patients, referred to as “airway remodelling”, are caused by persistent inflammation and subsequent inadequate repair of damaged airway epithelium [3-5].

Various cells are involved in cellular airway inflammation and subsequent airway remodelling, such as eosinophils, neutrophils, T lymphocytes and mast cells [4]. Infiltration of mast cells into the airway smooth muscle cell layer of allergic asthmatics is a key feature of asthma and is thought to be associated with AHR [6,7]. Upon mast cell degranulation various inflammatory mediators, such as vasoactive amines, cytokines and proteases are released [8,9]. Mouse mast cell protease 1 (mMCP-1) was reported to enhance airway narrowing in mice through effects on the epithelium [10,11].

Glucocorticoids (GCs) are by far the most effective treatments for asthma [12]. Besides the undesirable side effects of long-term GC therapy, a significant number of asthmatics is steroid resistant and fails to respond to this therapy [13,14]. These limitations of GCs therapy highlight the need for novel therapeutics with long-term benefits, greater disease control and increased efficacy.

Changes in the microbiota were reported to contribute to the development of allergies and asthma [15,16]. The potential role of beneficial bacteria, such as probiotics, as modulators of the intestinal microbiota and mucosal immune responses has been extensively investigated and discussed in the last few years [17-19]. Probiotics are “live microorganisms which, when consumed in adequate amounts, confer a health benefit on the host” [20,21]. *Bifidobacteria* and *Lactobacilli,* which are a part of the gut microbiota, were effective in suppressing both allergic and autoimmune responses, reducing allergic symptoms and inhibiting allergic airway response in murine models of acute airway inflammation [22-26].

Because they contain toll-like receptor (TLR) ligands, it is believed that beneficial bacteria can modulate TLR-driven responses and also skew the immune balance towards a Th1-associated response [27]. The TLRs and nod-like receptors (NLRs) are key pattern recognition receptor (PRR) families in the innate immune response. In the human airways, the RRRs are expressed in or on dendritic cells (DCs), epithelial cells, eosinophils, macrophages and mast cells [28,29]. Following TLR and NLR activation in the lung, various chemokines and cytokines are produced by eosinophils and mast cells that attract activated B-lymphocytes and Th lymphocytes to the lung to orchestrate the inflammation in the airways [30].

The function and expression of PRRs were linked to susceptibility towards allergic asthma [31,32]. Functional genetic variations in *TLR1*, *TLR10* and *TLR6* genes affecting gene and protein expression were associated with increased mRNA expression of these TLRs and protected against atopic asthma in humans [32]. Genetic variations in *TLR2, NOD1 and NOD2* genes that led to either decreased mRNA expression and affected microbial recognition, respectively, were positively associated with disease susceptibility and pathogenesis [25,33-36]. Cord blood CD34 (+) cells from high-atopic-risk infants have been reported to have low *TLR2*, *TLR4*, and *TLR9* expression and the latter was demonstrated to exert protective immunomodulatory effects on asthma [37-39]. *Tlr3* contributes to asthma exacerbations in mice [40] and a study in a murine macrophage cell line suggested a pro-inflammatory role of *Tlr4* and *5* in the disease [41].

In asthma, over 50 cytokines have now been identified to affect disease outcome. Strong pro-inflammatory and Th2-associated cytokines; including interleukin-1β (IL-1β), IL-4, IL-5, IL-6, IL-9, IL-13, IL-17, IL-25 and tumor necrosis factor α (TNF-α) were reported to enhance asthma [30]. On the other hand, Th1-associated cytokines; IL-12, IL-18 and interferon-γ (IFNγ) were reported to reduce the symptoms of the disease [30]. Additionally, subjects with asthma were reported to have reduced levels of the anti-inflammatory cytokine IL-10 in the sputum [42]. Moreover, Th1/Th2 imbalances as well as disturbed T helper type-17(Th17)/Treg balances were reported in asthmatic patients [43]. Imbalances in Th responses can also be detected using Th-specific transcription factors: T-bet for Th1 cells, GATA-3 for Th2 cells, retinoic acid orphan receptor-γt (RORγt) for Th17 cells and forkhead box P3 (Foxp3) for Tregs [44]. Alterations in the expression and/or function of Th-specific transcription factors were associated with asthma pathogenesis [45,46].

This study aimed to explore the therapeutic effects of long-term administration of *Bifidobacterium breve* M-16 V and *Lactobacillus rhamnosus* NutRes1 on chronic airway inflammation and remodelling in mice. A glucocorticoid was used as a reference treatment. Findings from this study will contribute to a better understanding of the immunomodulatory and therapeutic effects of beneficial bacteria in chronic allergic asthma.

## Methods

### Animals

Male BALB/c mice (6–8 weeks; Charles River Laboratories, France) were acclimated to their new environment for at least 1 week before the start of the experiment. Mice were housed under standard conditions and had free access to food and water. All *in vivo* experiments were approved by and were in accordance with the guidelines of the local Dutch Committee of Animal Experimentation.

### Chronic asthma model

#### OVA sensitisation

**Figure 1:**
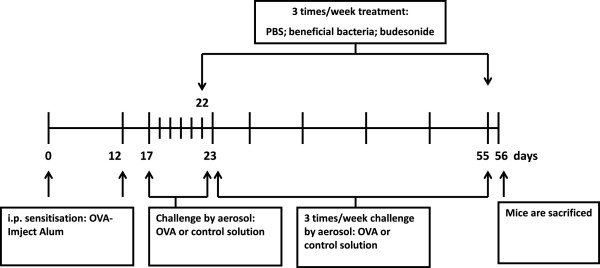
**Time schedule of the chronic asthma mouse model.** Male BALB/c mice were sensitised intraperitoneally to OVA-ImjectAlum on days 0 and 12 and mice were challenged from day 17 until day 23 daily with aerosolised OVA or saline. From day 22 until day 55, mice were treated 3 times a week with either PBS or budesonide by oropharyngeal aspiration or beneficial bacteria (*B. breve* or *L. rhamnosus*) by oral gavage. 1 h after treatment, from day 24 until day 55, mice were challenged 3 times/week with aerosolised OVA or saline. Mice were sacrificed on day 56 after pulmonary function measurement.

Sensitisations were performed on days 0 and 12*.* Mice were sensitised to OVA (chicken egg albumin, grade V, Sigma, St. Louis, MO, USA) by intraperitoneal injections of 0.1 mL alum-precipitated antigen, comprising 10 μg OVA absorbed into 2.25 mg alum (AlumImject; Pierce, Rockford, IL, USA). Control animals received 0.1 mL saline only (NaCl 0.9%; B. Braun Medical B.V., Oss, The Netherlands) (Figure 1).

#### OVA challenge

A chronic model of asthma was established according to a modification of a model of prolonged allergen-induced airway inflammation described in [2]. Mice were exposed daily to 5% OVA aerosol in saline or saline only using a Pari LC Star nebuliser (PARI GmbH, Starnberg, Germany) in an aerosol cabin for 20 min between days 17 and 23. Control animals were exposed to nebulised saline aerosol only. From day 24 until day 55, the frequency of challenge was reduced to three times a week and mice were exposed to aerosolised OVA (5%) or saline only for 20 min (Figure 1).

### Beneficial bacteria treatment

*Bifidobacterium breve* M-16 V (*B. breve*, Morinaga Milk Industry, Tokyo, Japan) and *Lactobacillus rhamnosus* NutRes1 (*L. rhamnosus*, Danone Research, Wageningen, the Netherlands) were grown in MRS (Oxoid, Basingstoke, UK), supplemented with 0.5 g/L L-cysteine for *Bifidobacteria*, at pH 6.5 and under anaerobic conditions. Bacteria were harvested in the early stationary phase, washed with phosphate buffered saline (PBS, Lonza Leusden, The Netherlands) and stored with glycerol 20% (w/v), in aliquots at -80°C. Cell counts were determined by plating serial dilutions and fluorescent microscopy by staining with DAPI. The bacteria were resuspended in PBS prior to use.

After development of airway inflammation, mice received 10^9^ colony forming units (CFUs) of *B. breve* or 1.1 × 10^9^ CFUs of *L. rhamnosus* per animal per day. Bacterial strains were suspended in 0.2 mL of PBS and given by oral gavage, 1 h prior to challenge, three times a week from day 22 until day 55 (Figure 1).

### Budesonide treatment

As a reference treatment, mice received 0.5 μg/g of mouse/day of budesonide (Sigma) in PBS. Budesonide was administered to mice by oropharyngeal aspiration after induction of light isoflurane anesthesia as described previously in [47], 1 h prior challenge, three times a week from day 22 until day 55. Control animals received 50 uL of PBS by the same administration route. Mice were rendered asthmatic following the schema presented in Figure 1.

### Airway response to methacholine

On day 56, 24 h after the last OVA aerosol challenge, the airway response to increasing doses of methacholine was analysed after insertion of a cannula in the trachea. Lung resistance in anesthetized, mechanically ventilated mice was measured directly using whole-body plethysmography (Emka technologies, Paris, France). Mice were exposed to saline (0 mg/mL methacholine) and increasing doses (0.38 to 25 mg/mL) of aerosolised methacholine (Sigma).

### Bronchoalveolar lavage

After sacrifice, on day 56, lungs were first lavaged through a tracheal cannula with 1 mL saline containing protease inhibitor cocktail (Complete Mini, Roche Diagnostics, Mannheim, Germany), pre-warmed at 37°C. This was followed by 3 additional lavages with 1 mL saline only. Cytospin cell preparations were made by cytospinning the cells onto glass for 5 min (400 *g,* 4°C) and cytospins were stained by DiffQuick (Merz & Dade AG, Düdingen, Switzerland). Numbers of eosinophils, macrophages, neutrophils and lymphocytes were scored by light microscopy.

### RNA isolation and quantitative real-time PCR

After mice were sacrificed on day 56, the lungs were dissected and mRNA was isolated from whole lung tissue. Messenger RNA isolation (n = 6 mice per group) was carried out according to the Qiagen RNeasy Mini Kit protocol (Qiagen Benelux B.V., Venlo, The Netherlands). Reverse transcriptase PCR was performed using an iScript™cDNA Synthesis Kit (Bio-Rad Laboratories, Hercules, CA, USA). The reactions were performed in a PTC-100TM Programmable Thermal Controller (M. J. Research Inc., Waltham, Massachusetts, USA) according to manufacturer’s protocol.

cDNA was amplified using iQ SYBR Green supermix in a 96-well PCR plate and run in a CFX96 Real-Time PCR Detection System (Bio-Rad). Primers for TLRs, NLRs, ribosomal protein S13 (RPS13, reference gene) and T cell transcription factors were purchased by Isogen (Isogen Life Science, De Meern, The Netherlands). The sequences are listed in Additional file 1: Table S1. For mouse T cell cytokines, RT^2^ qPCR Primer Assays (SABiosciences, Venlo, The Netherlands) were used. The protocol used for amplification was 94°C for 3 min, 94°C for 10 sec, specific melt temperature for 45 sec, followed by 39 cycles of 94°C for 10 sec and 95°C for 10 sec.

Normalised gene expression (∆∆C_T_) was calculated using the built-in gene expression analysis module in CFX Manager™ software (CFX Manager™ software version 1.6).

### Foxp3 staining and flow cytometry

On days 0 and 56, blood samples were collected from mice by cardiac puncture in tubes containing lithium heparin to prevent coagulation. The blood was then washed in PBS and, after centrifugation; the pellet was subjected to red cell lysis using a buffer containing NH_4_Cl (MERCK, Darmstadt Germany), KHCO_3_ (Sigma), ethylenediaminetetraacetic acid (EDTA, MERCK) in demineralised water for 5 min on ice. After several washes with PBA (PBS containing 1% bovine serum albumin (BSA, Roche Diagnostics, Almere, The Netherlands) cells were resuspended in PBA and kept on ice until Foxp3 staining.

The expression of Foxp3 was measured using the Foxp3 Staining Buffer Set (eBioscience, San Diego, CA, USA) using the following protocol: cells were incubated in Fixation/Permeabilization buffer for 30 min on ice. Cells were then washed once with PBA followed by two washes with permeabilization buffer. After a 15 min preincubation in total mouse serum blocking reagent on ice, cells were washed once with permeabilization buffer and then stained with anti-CD4 (FITC, eBioscience), anti-CD25 (PE, eBioscience) and anti-Foxp3 (APC, eBioscience) for 30 min on ice. Cells were washed twice with permeabilization buffer and resuspended in PBA for flow cytometry analysis.

Tregs were defined as CD4 + Foxp3 + CD25hi T cells. The stained cells were analysed on a FACSCanto II flow cytometer (BD Biosciences, USA). Data analysis was performed using BD FACSDiva™ software (BD Biosciences). The gating strategy is illustrated in Additional file 2: Figure S1.

### Measurement of mouse mast cell protease 1 levels in serum

To assess mast cell activation, after mice were sacrificed on day 56, blood samples were collected from mice by cardiac puncture. The blood was coagulated for 1 h at room temperature and subsequently centrifuged for 5 min at 17,500 *g*. Serum samples were stored at -80°C until further analysis. Mouse mast cell protease 1 (mMCP-1) protein expression levels in serum were determined by enzyme-linked immunosorbent assay (ELISA) using the Mouse MCPT-1 (mMCP-1) ELISA Ready-SET-Go!® kit (eBioscience) according to manufacturer’s protocol.

### Measurement of cytokine production by T cells in thoracic lymph nodes after restimulation with anti-CD3 antibody *in vitro*

In order to examine specific T cell responses, after mice were sacrificed on day 56, lung-draining lymph nodes were collected from the thorax and transferred to cold sterile PBS. Single cell suspensions of the thoracic lymph nodes (TLNs) were made using a 70 μm nylon cell strainer (BD Biosciences) and rinsed with 15 mL of PBS. The cells were washed and resuspended in RPMI 1640 culture medium without L-glutamine and phenol red (Lonza) supplemented with 10% heat-inactivated fetal calf serum (FCS, Hyclone Laboratories, USA) and 0.1% penicillin-streptomycin solution (pen-strep, Sigma). The total number of cells was determined using a Beckman Z1 coulter® Particle Counter (Beckman, USA). TLN cells (4 × 10^6^ cells/mL) were cultured in a Greiner bio-one CellSTAR 96-well U-bottom plate (Greiner Bio-One B.V., Alphen a/d Rijn, The Netherlands) in medium with or without O/N pre-coating of the wells with 50 μg/mL of anti-CD3 antibody (Bioceros BV, Utrecht, The Netherlands). The supernatant was harvested after 5 days of culture at 37°C in 5% CO2 and stored at -20°C until further analysis.

The levels of cytokines in the supernatant were measured by flow cytometry using a BD™ Cytometric Bead Array (CBA) Mouse Th1/Th2/Th17 Cytokine kit (BD Biosciences) according to manufacturer’s protocol on a FACSCanto II flow cytometer (BD Biosciences). Data analysis was performed using BD FCAP Array™ v3.0.1 Software (BD Bisosciences).

### Histology and immunohistochemistry

After mice were sacrificed on day 56, lungs were fixed with 10% formalin infusion through a tracheal cannula at a constant pressure of 25 cm H_2_O. The lungs were immersed in fixative for at least 24 h, after which the left lung was embedded in paraffin. After paraffin embedding, 5 μm sections were cut and de paraffin sections were first deparaffinised. Endogenous peroxidase activity was blocked with 0.3% H_2_O_2_ (Merck) in methanol for 30 min at room temperature and rehydrated in a graded ethanol series to PBS and paraffin section were stained with hematoxylin/eosin (H&E) for inflammation, periodic acid-schiff (PAS) for goblet cells, Masson’s trichrome for connective tissue, rabbit polyclonal anti-α-smooth muscle actin antibody (Abcam, Cambridge, UK) for smooth muscle cells and rabbit polyclonal anti-Ki76 antibody (Abcam) for proliferating cells according to standard methods. Photomicrographs were taken with an Olympus BX50 microscope equipped with a Leica DFC 320 digital Camera.

Slides were reviewed in blinded fashion by two observers independently and slides were scored on the basis of the percentage of positive stained cells in the following way: -, no positive staining; +/-, less than 25% of cells stained positive; +, 25 to 50% cells stained positive; ++, 50 to 75% cells stained positive.

### Statistical analysis

Data analysis was performed using a 1-way analysis of variance (one-way ANOVA) with the Bonferroni’s post-hoc test. Linear regression analysis was used to calculate correlations. All statistical analyses were performed using GraphPad Prism software program (GraphPad Prism software version 5.03).

## Results

### *L. rhamnosus* and budesonide reduce the increased lung resistance in chronically asthmatic mice

To investigate the lung function in the chronic asthmatic mice after treatments, lung resistance was measured (Table 1). OVA-sensitised and challenged control mice (OVA/OVA-PBS) showed a significant increase in the basal lung resistance and lung resistance at all concentrations of methacholine as compared to the sensitised only control group (OVA/Sal-PBS). Treatment of sensitised and challenged mice with *L. rhamnosus* (OVA/OVA-*L. rhamnosus*) resulted in a significant decrease in the basal lung resistance and lung resistance at 0, 0.75 and 1.56 mg/mL methacholine as compared to the OVA/OVA-PBS group. Budesonide treatment significantly decreased the basal lung resistance and lung resistance at 0, 0.38, 0.75 and 1.56 mg/mL methacholine in allergic mice (OVA/OVA-BUD) as compared to OVA/OVA-PBS mice. The basal lung resistance and lung resistance at all concentrations of methacholine remained unchanged in *B. breve-*treated*,* sensitised and challenged mice (OVA-OVA-*B. breve*) as compared to the OVA/OVA-PBS group.

**Table 1:** ***L. rhamnosus *** **and budesonide treatment reduce the basal lung resistance in chronic asthmatic mice**

**Group**	**Methacholine (mg/mL)**																	
	**Basal**		**0 (saline)**		**0.38**		**0.75**		**1.56**		**3.13**		**6.25**		**12.5**		**25**	
	**Mean**	**SEM**	**Mean**	**SEM**	**Mean**	**SEM**	**Mean**	**SEM**	**Mean**	**SEM**	**Mean**	**SEM**	**Mean**	**SEM**	**Mean**	**SEM**	**Mean**	**SEM**
OVA/Sal-PBS	0.73	0.09	0.76	0.09	0.80	0.06	0.88	0.06	0.97	0.08	1.04	0.09	1.16	0.06	1.23	0.07	1.41	0.12
OVA/OVA-PBS	1.81^#^	0.31	1.79^#^	0.22	1.76^#^	0.19	1.85^#^	0.20	1.86^#^	0.21	1.82^#^	0.19	1.73^#^	0.15	1.96^#^	0.18	2.01^#^	0.21
OVA/Sal-*B. breve*	0.61	0.09	0.76	0.15	0.93	0.21	0.96	0.17	0.97	0.13	1.00	0.07	1.12	0.09	1.17	0.09	1.16	0.06
OVA/OVA-*B. breve*	1.74	0.58	1.66^¤^	0.59	1.54	0.43	1.55	0.33	1.66	0.28	1.86	0.30	1.97	0.27	2.18	0.29	2.36	0.30
OVA/Sal-*L. rhamnosus*	0.64	0.09	0.73^#^	0.16	0.74	0.09	0.81	0.11	0.83	0.08	0.93	0.08	1.02	0.10	1.05	0.08	1.20	0.12
OVA/OVA-*L. rhamnosus*	1.05^¤^	0.18	1.15^¤^	0.14	1.13	0.13	1.18^¤^	0.13	1.30^¤^	0.12	1.48	0.13	1.55	0.14	1.69	0.16	1.80	0.16
OVA/Sal-BUD	0.55	0.02	0.56^#^	0.02	0.63	0.04	0.71	0.07	0.79	0.09	0.85	0.10	0.96	0.09	1.08	0.12	1.17	0.11
OVA/OVA-BUD	0.85^¤^	0.12	0.88^¤^	0.09	1.01^¤^	0.09	1.10^¤^	0.09	1.23^¤^	0.13	1.38	0.13	1.57	0.15	1.73	0.10	1.80	0.16

Airway response to saline and increasing doses of methacholine measured 24 h after the last OVA or saline challenge as expressed by lung resistance (LR). Basal airway response is also shown. Mice in all groups were sensitised to OVA. OVA/Sal mice were challenged with saline; OVA/OVA mice were challenged with OVA. Mice were treated with PBS (OVA/Sal-PBS; OVA/OVA-PBS), *B. breve* (OVA/Sal-*B. breve*; OVA/OVA-*B. breve*), *L. rhamnosus* (OVA/Sal-*L. rhamnosus*; OVA/OVA-*L. rhamnosus*) or budesonide (OVA/Sal-budesonide; OVA/OVA-budesonide). Results are shown as mean ± SEM, n = 6 mice/group. Statistical significance of differences was tested using post hoc Bonferroni’s multiple comparison test after one-way ANOVA. ^**#**^Statistically significant difference (p < 0.05) compared to OVA/Sal-PBS mice. ^**¤**^Statistically significant difference (p < 0.05) compared to OVA/OVA-PBS mice. BUD = budesonide.

### *B. breve* and *L. rhamnosus* are as effective as budesonide in reducing pulmonary inflammation in chronically asthmatic mice

**Figure 2:**
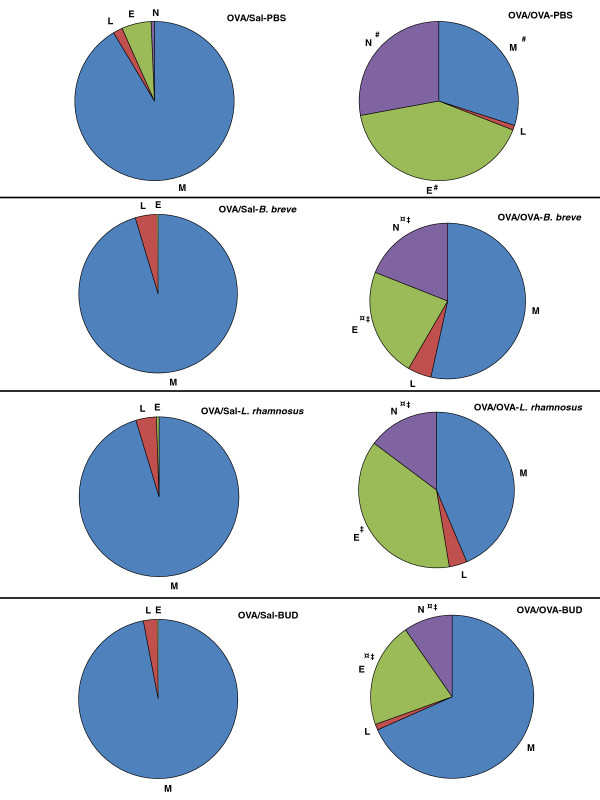
**Beneficial bacteria and budesonide reduce the differential BAL fluid cell counts in chronic asthmatic mice.** Pulmonary inflammation is represented by the influx of macrophages (M), lymphocytes (L), eosinophils (E) and neutrophils (N) in the BAL fluid. Differential cell counts are shown as percentages of the total cell count for each group. The charts show the mean, n = 6 mice/group. Statistical significance of differences was tested using post hoc Bonferroni’s multiple comparison test after one-way ANOVA. ^#^Statistically significant difference (p < 0.05) compared to OVA/Sal-PBS mice. ^¤^Statistically significant difference (p < 0.05) compared to OVA/OVA-PBS mice. ^‡^Statistically significant difference (p < 0.05) compared to the OVA/Sal group in each treatment group. BUD = budesonide.

To gauge the extent of inflammation in the asthmatic mice after treatments, bronchoalveolar lavage (BAL) fluid was examined for leukocyte accumulation (Figure 2). OVA/OVA-PBS mice showed a significant increase in the total inflammatory cell number (155.25 × 10^4^ ± 12.58; n = 6; p < 0.0001) in the BAL fluid, which was due to a relative increase in the number of macrophages, eosinophils and neutrophils as compared to OVA/Sal-PBS mice (29.25 × 10^4^ ± 3.12; n = 6). All treatments significantly reduced the total number of inflammatory cells in the BAL fluid of OVA/OVA mice. Budesonide treatment significantly decreased the total inflammatory cell number (83.70 × 10^4^ ± 13.66; n = 6; p < 0.0001) and relative number of eosinophils and neutrophils in OVA/OVA-BUD mice. *B. breve* treatment, however, further decreased the total inflammatory cell number (73.50 × 10^4^ ± 6.42, n = 6; p < 0.0001) and significantly decreased the relative number of eosinophils and neutrophils. *L. rhamnosus* treatment significantly decreased the total inflammatory cell number (79.75 × 10^4^ ± 17.49; n = 6; p < 0.0001) and relative number of neutrophils.

### *B. breve* and *L. rhamnosus* modulate TLR and NLR mRNA expression in lung tissue of chronically asthmatic mice

**Figure 3:**
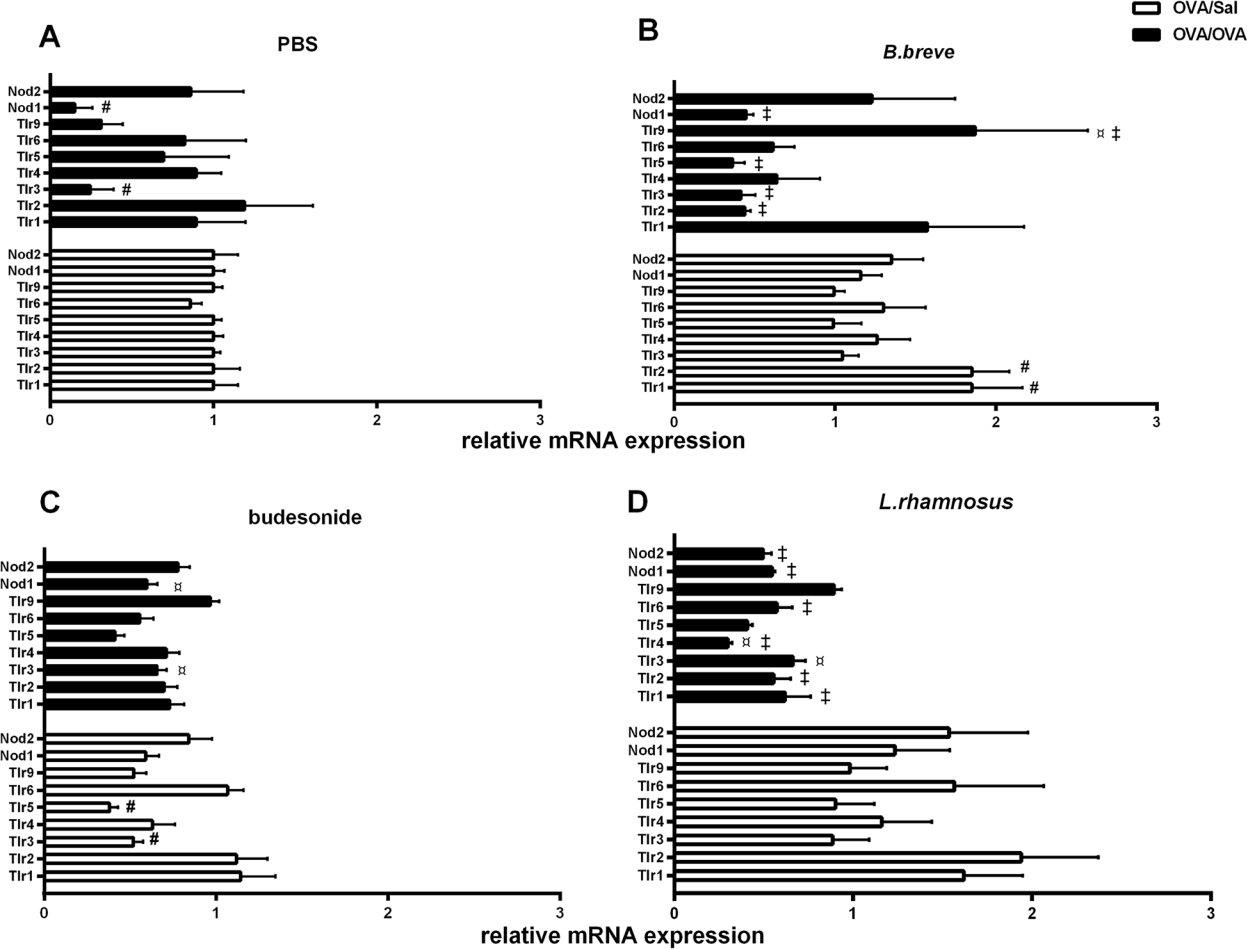
**Beneficial bacteria modulate TLR and NLR mRNA expression in lung tissue of chronically asthmatic mice.** Data is shown as mean ± SEM, n = 6 mice/group, of the TLR and NLR mRNA expression levels in OVA-sensitised, Sal-challenged (OVA/Sal; white bars) mice and OVA-sensitised, OVA-challenged (OVA/OVA; black bars) mice treated with PBS **(A)**, *B. breve***(B)**, budesonide **(C)** or *L. rhamnosus***(D)**. The results are presented as mRNA expression levels relative to levels found in the OVA/Sal-PBS mice (white bars in figure A). Statistical significance of differences was tested using the post hoc Bonferroni’s multiple comparison test after one-way ANOVA. ^#^Statistically significant difference (p < 0.05) compared to the OVA/Sal-PBS group. ^¤^Statistically significant difference (p < 0.05) compared to the OVA/OVA-PBS group. ^‡^Statistically significant difference (p < 0.05) compared to the OVA/Sal group in each treatment group.

As PRRs in the lung can modulate ongoing chronic inflammation during asthma, the mRNA expression of *Tlr1-9* and *Nod1-2* were measured (Figure 3). The mRNA expression of *Tlr3* and *Nod1* was significantly decreased and *Tlr9* was also decreased (p > 0.05) in OVA/OVA-PBS mice as compared to the OVA/Sal-PBS group (Figure 3A). After *B. breve* treatment, the OVA/OVA-*B. breve* mice showed a significant increase in *Tlr9* expression compared to the OVA/OVA-PBS group. In non-asthmatic controls, *Tlr1 and Tlr2* expression was significantly increased in the OVA-Sal-*B. breve* group as compared to OVA/Sal-PBS mice, yet, the expression of the other PRRs in non-asthmatic controls remained unchanged. *B.breve* treatment in asthmatic mice significantly increased *Tlr9* expression and decreased *Tlr2, Tlr3, Tlr5* and *Nod1* expression in the OVA/OVA-*B. breve* group as compared to the OVA-Sal-*B. breve* group (Figure 3B). Mice in the OVA/OVA-budesonide group showed significant increases in the expression of *Tlr3* and *Nod1* and *Tlr9* expression was higher (p > 0.05) as compared to the OVA/OVA-PBS group. The expression of *Tlr3* and *Tlr5* was significantly decreased in OVA/Sal-budesonide mice as compared to the OVA/Sal-PBS group (Figure 3C). The OVA/OVA-*L. rhamnosus* group showed a significant decrease in *Tlr4*, an increase in *Tlr3* and higher *Tlr9* expression compared to OVA/OVA-PBS mice. The mRNA expression of *Tlr1*, *Tlr2*, *Tlr4*, *Tlr6, Nod1* and *Nod2* was significantly decreased in OVA/OVA- *L. rhamnosus* mice as compared to the OVA/Sal-*L. rhamnosus* group (Figure 3D).

### Beneficial bacteria and budesonide treatment modulate cytokine mRNA expression in lung tissue of chronically asthmatic mice

**Figure 4:**
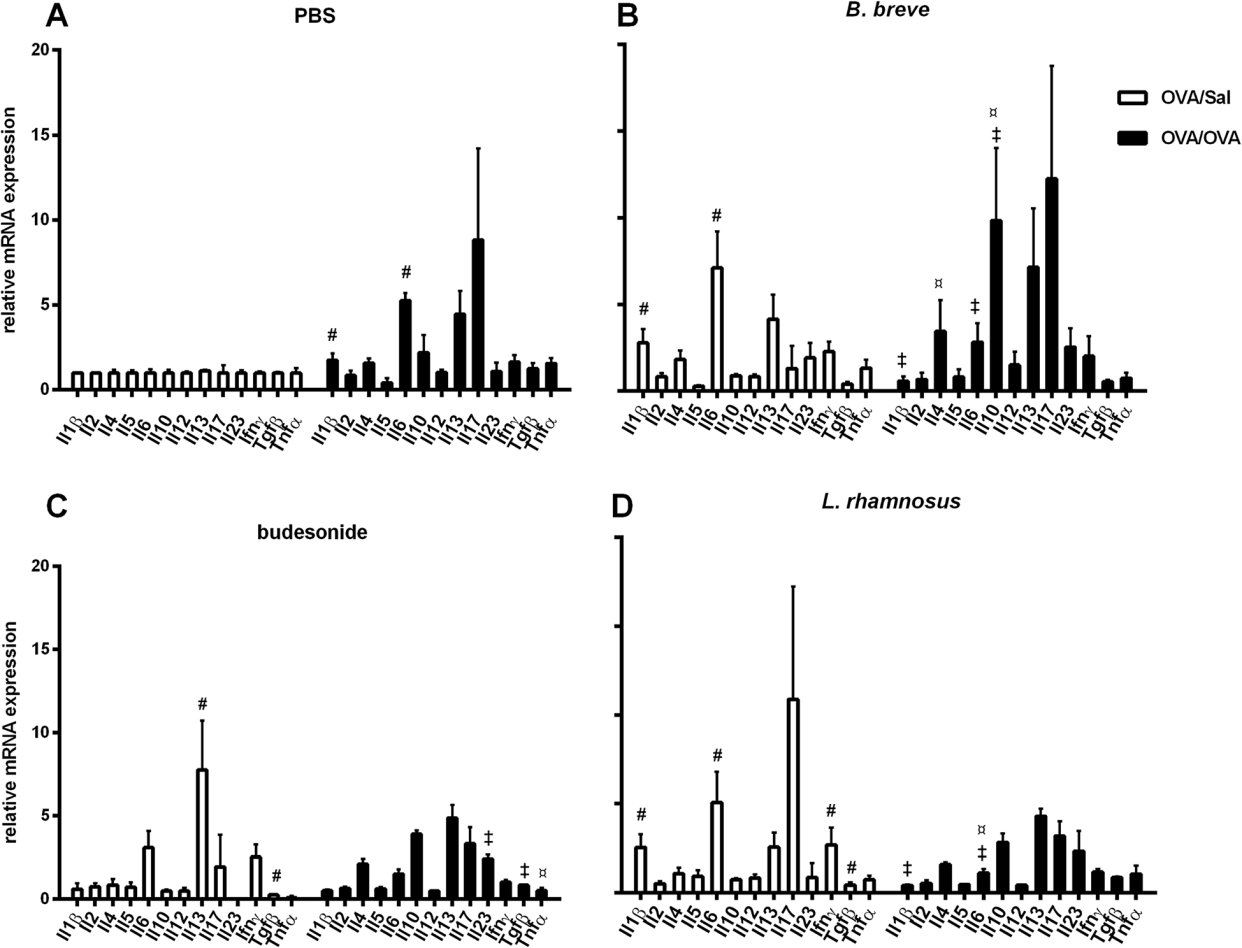
**Beneficial bacteria and budesonide modulate cytokine mRNA expression in lung tissue of chronically asthmatic mice.** Data is shown as mean ± SEM, n = 6 mice/group, of the cytokine mRNA expression levels in OVA-sensitised, Sal-challenged (OVA/Sal; white bars) mice and OVA-sensitised, OVA-challenged (OVA/OVA; black bars) mice treated with PBS **(A)**, *B. breve***(B)**, budesonide **(C)** or *L. rhamnosus***(D)**. The results are presented as mRNA expression levels relative to levels found in the OVA/Sal-PBS mice (white bars in figure A). Statistical significance of differences was tested using the post hoc Bonferroni’s multiple comparison test after one-way ANOVA. ^#^Statistically significant difference (p < 0.05) compared to the OVA/Sal-PBS group. ^¤^Statistically significant difference (p < 0.05) compared to the OVA/OVA-PBS group. ^‡^Statistically significant difference (p < 0.05) compared to the OVA/Sal group in each treatment group.

To determine the extent of inflammation and the Th response in the lung, the mRNA expression of various cytokines was measured (Figure 4). The mRNA expression of *Il1β* and *Il6* was significantly increased and *Il13* and *Il17* expression was also increased (p > 0.05) in OVA/OVA-PBS mice as compared to the OVA/Sal-PBS group (Figure 4A). The OVA/OVA*-B. breve* group showed a significant increase in *Il4* and *Il10* expression as compared to the OVA/OVA-PBS group. Expression of *Il1β* and *Il6* was significantly increased in OVA/Sal-*B. breve* mice as compared to the OVA/Sal-PBS group. The mRNA expression of these two cytokines was significantly decreased in OVA/OVA*-B. breve* mice as compared to the OVA/Sal-*B. breve* group (Figure 4B). The OVA/OVA-budesonide group showed a significant decrease in the expression of *Tnfα* as compared to OVA/OVA-PBS mice. Expression of *Il13* was significantly increased and *Tgfβ* expression was significantly decreased in OVA/Sal-budesonide mice as compared to the OVA/Sal-PBS group. The mRNA expression of *Il23* and *Tgfβ* was significantly increased in OVA/OVA-budesonide mice as compared to the OVA/Sal-budesonide group (Figure 4C). *L. rhamnosus* treatment significantly decreased the expression of *Il6* in OVA/OVA-*L. rhamnosus* mice as compared to the OVA/OVA-PBS group. The expression of *Il1β, Il6* and *Ifnγ was* significantly increased and *Tgfβ* expression was significantly decreased in OVA/Sal-*L. rhamnosus* mice compared to the OVA/Sal-PBS group. The mRNA expression of *Il1β* and *Il6* was significantly decreased in OVA/OVA-*L. rhamnosus* mice as compared to the OVA/Sal-*L. rhamnosus* group (Figure 4D)*.*

### *B. breve* treatment results in a strong up-regulation of mRNA for *Tbet* and *Foxp3* and relevant Th cytokines mRNA expression in the lungs of chronically asthmatic mice

**Figure 5:**
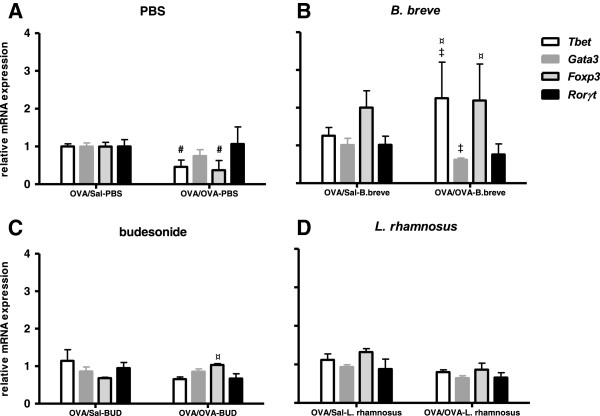
***B. breve *treatment up-regulates Tbet and Foxp3 mRNA expression in lungs of chronically asthmatic mice.** Data is shown as mean ± SEM, n = 6 mice/group, of the transcription factor mRNA expression levels in OVA-sensitised, Sal-challenged (OVA/Sal) mice and OVA-sensitised, OVA-challenged (OVA/OVA) mice treated with PBS **(A)**, *B. breve***(B)**, budesonide **(C)** or *L. rhamnosus***(D)**. The results are presented as mRNA expression levels relative to levels found in the OVA/Sal-PBS mice (white bars in figure A). Statistical significance of differences was tested using the post hoc Bonferroni’s multiple comparison test after one-way ANOVA. ^#^Statistically significant difference (p < 0.05) compared to the OVA/Sal-PBS group. ^¤^Statistically significant difference (p < 0.05) compared to the OVA/OVA-PBS group. ^‡^Statistically significant difference (p < 0.05) compared to the OVA/Sal group in each treatment group.

To further explore the effects of the different treatments on Th responses in the lung, the mRNA expression of Th-specific transcription factors was measured (Figure 5). The expression of Th1-(*Tbet*) and Treg-(*Foxp3*) transcription factors was significantly decreased in the OVA/OVA-PBS group as compared to the OVA/Sal-PBS group. No correlations were found between the expression of these transcription factors and relevant Th cytokines. The expression of Th2-(*Gata3*), and Th17-(*Rorγt*) transcription factors remained unchanged (Figure 5A). However, *Tbet* and *Foxp3* expression was significantly increased in OVA/OVA-*B*. *breve* mice as compared to the OVA/OVA-PBS group. Interestingly, expression of *Tbet* was tightly correlated with *Ifnγ* (R^2^ = 0.903; p = 0.013) and *Il12* (R^2^ = 0.994; p = 0.003) and expression of *Foxp3* was tightly correlated with *Il10* (R^2^ = 0.860; p = 0.024) expression in whole lung tissue of OVA/OVA-*B. breve* mice. Compared to the OVA/Sal-PBS group, *Foxp3* expression was almost two-fold increased (P > 0.05) in the OVA/Sal-*B. breve* group. Expression of *Tbet* was significantly increased and expression of *Gata3* was significantly decreased in OVA/OVA-*B*. *breve* mice as compared to the OVA/Sal-*B. breve* group (Figure 5B). A significant increase in the expression of *Foxp3* was observed in the OVA/OVA-BUD group as compared to OVA/OVA-PBS mice (Figure 5C). The expression of all Th-specific transcription factors remained unchanged in the OVA/Sal-*L. rhamnosus* and OVA/OVA-*L. rhamnosus* groups (Figure 5D). Expression of *Foxp3,* however*,* was tightly correlated with *Il10* expression (R^2^ = 0.926; p = 0.009) in OVA/OVA-*L. rhamnosus* mice*.* None of the test groups showed correlations between the expression of *Gata3* and Th2-related cytokines (*Il4, Il5, Il13*) and *Rorγt* and Th17-related cytokines (*Il17, Il23*).

### *B. breve* skews the immune response away from Th2 and towards Treg in the lungs of chronically asthmatic mice

To determine the extent of Th response skewing in the lung, ratios for *Gata3/Tbet* (Th2/Th1), *Foxp3/Rorγt* (Treg/Th17)*, Foxp3/Gata3* (Treg/Th2*)* and *Foxp3/Tbet* (Treg/Th1) mRNA expression were calculated (Table 2). The OVA/OVA-PBS groups showed a Th2-skewed immune response represented by a significant increase in *Gata3/Tbet* ratio as compared to OVA/Sal-PBS mice. This ratio was significantly decreased in the OVA/OVA-*L. rhamnosus* and OVA/OVA-*B. breve* groups, but not the OVA/OVA-budesonide group. The Th2-reducing effect of OVA-OVA-*B. breve* was even more effective in challenged mice than healthy mice as the *Gata3/Tbet* ratio was decreased in OVA/OVA-*B. breve* mice as compared to the OVA/Sal-*B. breve* group*.* This was not the case for budesonide. Compared to the OVA/Sal-budesonide group, there was a significant increase in the *Gata3/Tbet* ratio in the OVA/OVA-budesonide group. Interestingly, the *Foxp3/Rorγt* and *Foxp3/Gata3* ratios were significantly increased in OVA/OVA-*B. breve* mice as compared to the OVA/OVA-PBS group indicating an increase in Treg-associated responses. The ratio of *Foxp3/Tbet* did not differ significantly among the different treatment groups.

**Table 2:** ***B. breve*** **skews the immune response away from Th2 and towards Treg in lungs of chronically asthmatic mice**

**Treatment**	**Ratio**	**OVA/Sal**		**OVA/OVA**	
		**Mean ratio**	**SEM**	**Mean ratio**	**SEM**
PBS	*Gata3/Tbet*	1.03	0.10	1.66^#^	0.37
	*Foxp3/Rorγt*	0.82	0.09	0.29	0.20
	*Foxp3/Gata3*	0.83	0.09	0.41	0.28
	*Foxp3/Tbet*	0.86	0.09	0.69	0.47
*B. breve*	*Gata3/Tbet*	0.83	0.14	0.29^¤, ‡^	0.02
	*Foxp3/Rorγt*	1.62	0.36	2.36^¤^	1.04
	*Foxp3/Gata3*	1.65	0.36	2.92^¤^	1.29
	*Foxp3/Tbet*	1.36	0.30	0.83	0.37
budesonide	*Gata3/Tbet*	0.78	0.10	1.34^‡^	0.11
	*Foxp3/Rorγt*	0.59	0.02	1.26	0.04
	*Foxp3/Gata3*	0.66	0.02	1.00	0.03
	*Foxp3/Tbet*	0.51	0.02	1.35	0.04
*L. rhamnosus*	*Gata3/Tbet*	0.86	0.06	0.83^¤^	0.08
	*Foxp3/Rorγt*	1.02	0.22	1.07	0.21
	*Foxp3/Gata3*	0.98	0.21	1.11	0.20
	*Foxp3/Tbet*	0.84	0.18	0.92	0.18

Ratios for Gata3/Tbet (Th2/Th1), Foxp3/Rorγt (Treg/Th17), Foxp3/Gata3 (Treg/Th2) and Foxp3/Tbet (Treg/Th1) mRNA expression in whole lung tissue are shown for OVA/Sal and OVA/OVA mice for each treatment. Data is represented as mean ratio ± SEM, n = 6 mice/group. The mean ratio was calculated by dividing the individual expression values for the first transcription factor (numerator) by the mean expression value for the second transcription factor (denominator). Statistical significance of differences was tested using post hoc Bonferroni’s multiple comparison test after one-way ANOVA. ^**#**^Statistically significant difference (p < 0.05) compared to the OVA/Sal-PBS group. ^**¤**^Statistically significant difference (p < 0.05) compared to the OVA/OVA-PBS group. ^**‡**^Statistically significant difference (p < 0.05) compared to OVA/Sal mice in each treatment group.

### Beneficial bacteria and budesonide increase Foxp3 expression in blood Treg cells of chronically asthmatic mice

**Figure 6:**
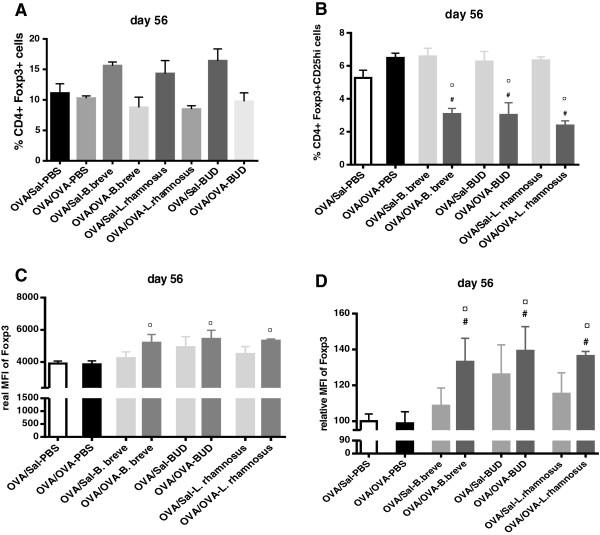
**Beneficial bacteria and budesonide increase Foxp3 expression in blood Treg cells of chronically asthmatic mice.** T cells were isolated on day 56 from mouse whole blood from OVA-sensitised, Sal-challenged (OVA/Sal) mice and OVA-sensitised, OVA-challenged (OVA/OVA) mice for each treatment. **(A)** Percentage of CD4 cells that are Foxp3+. **(B)** Percentage of CD4 cells that are Foxp3+ and CD25hi. **(C)** Real mean fluorescence intensity (MFI) of Foxp3 in Treg cells. **(D)** MFI levels relative to the OVA/Sal-PBS group (white bar). Data is shown as mean ± SEM, n = 6 mice/group. Statistical significance of differences was tested using the post hoc Bonferroni’s multiple comparison test after one-way ANOVA. ^#^Statistically significant difference (p < 0.05) compared to the OVA/Sal-PBS group. ^¤^Statistically significant difference (p < 0.05) compared to the OVA/OVA-PBS group. BUD = budesonide.

In order to determine if the transcriptional changes induced in *Foxp3* expression could be found at a protein expression level, flow cytometry was employed to examine Foxp3 expression on Treg cells in the blood (Figure 6). Treg cells were identified as CD4+ Foxp3 + CD25hi T cells and the magnitude of Foxp3 expression on each cell was determined by examining the mean fluorescence intensity (MFI) of the Foxp3 staining. The percentage of CD4 cells that are Foxp3+ did not differ among the different groups (Figure 6A). The percentage of CD4 cells that are Foxp3+ and CD25hi was significantly decreased by all treatments as compared to both OVA/Sal-PBS and OVA/OVA-PBS groups (Figure 6B). However, all treatments increased the Foxp3 expression in the blood Treg cells. The staining intensity of Foxp3 on day 56 was significantly higher in the OVA/OVA-*B. breve*, OVA/OVA-BUD and OVA/OVA-*L. rhamnosus* groups as compared to OVA/OVA-PBS mice and to the OVA/Sal-PBS group (Figure 6C and D). The staining intensity of Foxp3 on day 0 (before sensitisation, challenge and treatment) did not differ among the different groups (data not shown).

### *B. breve* and *L. rhamnosus* are as effective as budesonide in suppressing mucosal mast cell degranulation in chronically asthmatic mice

**Figure 7:**
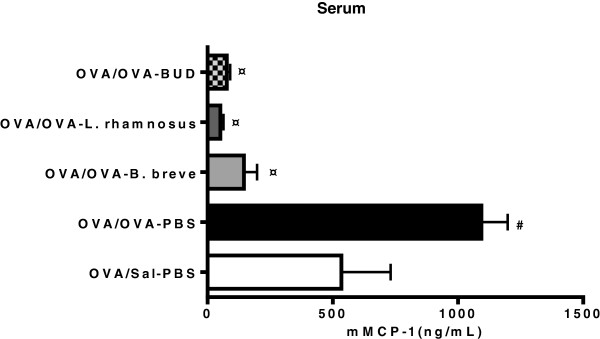
**Beneficial bacteria and budesonide reduce mouse mast cell protease-1 levels in serum of chronic asthmatic mice.** Mouse serum was isolated on day 56 and mouse mast cell protease-1 (mMCP-1) levels were measured by ELISA. Data is shown as mean ± SEM, n = 5-6mice/group. ^#^Statistically significant difference (p < 0.05) compared to the OVA/Sal-PBS group. ^¤^Statistically significant difference (p < 0.05) compared to the OVA/OVA-PBS group. BUD = budesonide.

To examine the effect of the different treatments on mast cells activity, enzyme-linked immunosorbent assay was employed to measure mMCP-1 protein levels in serum (Figure 7). The expression of mMCP-1 was significantly increased in OVA/OVA-PBS mice as compared to the OVA/Sal-PBS group. All treatments were effective in suppressing mucosal mast cell degranulation in OVA/OVA mice, as depicted by decreased protein levels of mMCP-1 in serum when compared to the OVA/OVA-PBS group. mMCP-1 levels in control animals (OVA/Sal) did not change after administration of beneficial bacteria (data not shown).

### Beneficial bacteria and budesonide reduce T cell activity in chronically asthmatic mice

**Figure 8:**
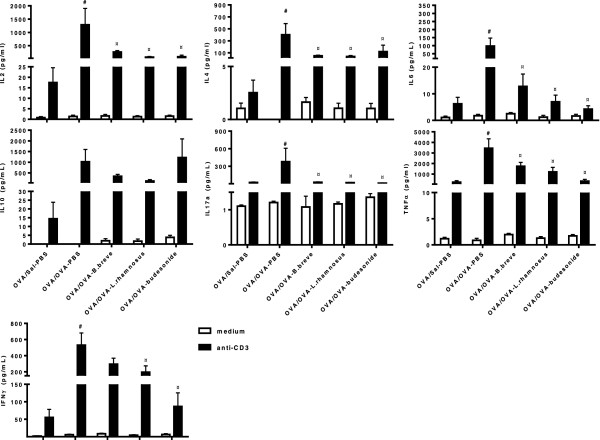
**Beneficial bacteria and budesonide suppress cytokine production by T cells in TLNs of chronic asthmatic mice.** Thoracic lymph nodes (TLNs) were isolated from mice on day 56 and restimulated in vitro with plate-bound anti-CD3 monoclonal antibody or medium only for 5 days (37°C, 5% CO_2_). Data is shown as mean of cytokine concentration (ng/ml) ± SEM, n = 6 mice/group. Statistical significance of differences was tested using post hoc Bonferroni’s multiple comparison test after one-way ANOVA. ^#^Statistically significant difference (p < 0.05) compared to the OVA/Sal-PBS group. ^¤^Statistically significant difference (p < 0.05) compared to the OVA/OVA-PBS group.

To investigate the effect of the treatments on cytokine production by T cells in lung draining lymph nodes, TLN-cell cultures were stimulated with anti-CD3, a pan T cell stimulator, and the cytokine levels in the supernatant were measured (Figure 8). Cytokine expression levels in medium-stimulated TLN cells did not differ among the different groups. The levels of IL-2, IL-4, IL-6, IL-17a, TNF-α and IFN-γ were significantly increased in anti-CD3-stimulated TLN cells of OVA/OVA-PBS mice as compared to samples from the OVA/Sal-PBS group. All treatments were effective in inhibiting T cell activity in OVA/OVA mice, significantly decreasing the levels of IL-2, IL-4, IL-6, IL-17a and TNF-α as compared to the OVA/OVA-PBS group. Additionally, *L. rhamnosus* and budesonide treatment significantly decreased the levels of IFN-γ in OVA/OVA mice as compared to the OVA/OVA-PBS group. IL-10 levels were increased (p > 0.05) in the OVA/OVA-PBS and in all treatment groups as compared to the OVA/Sal-PBS group. No differences in cytokine expression were observed in the TLNs of the different control groups (data not shown).

### *B. breve* and *L. rhamnosus* are as effective as budesonide in suppressing airway remodelling features in chronically asthmatic mice

**Figure 9:**
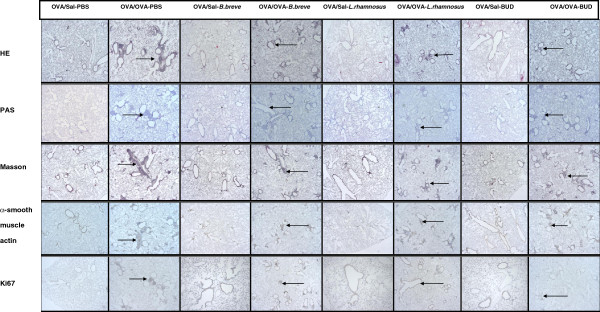
**Beneficial bacteria and budesonide suppress airway remodelling features in chronic asthmatic mice.** Representative photomicrographs for 5 mice/group of hitstological and immunohistochemical staining for inflammation (hematoxylin-eosin, HE); goblet cells (periodic acid Schiff, PAS); connective tissue (Masson trichrome, Masson); Anti-α-smooth muscle actin (α-smooth muscle) and proliferating cells (Anti-Ki67) in lung tissue of sensitised only (OVA/Sal) and sensitised and challenged (OVA/OVA) mice treated with PBS, *B. breve*, *L. rhamnosus* or budesonide (BUD). Original magnification, 40X, except for anti-Ki67 100X.

In order to examine the effect of the different treatments on airway remodelling features, semi-quantivitive histological and immunhistochemical analyses of lung tissue were performed (Figure 9 and Table 3). In the lung sections of OVA/OVA-PBS mice, increased inflammation score and number of goblet cells, collagenous connective tissue fibers, airway smooth muscle cells and proliferating cells was observed as compared to the OVA/Sal-PBS group. All treatments were effective in reducing the inflammation score and decreasing the number of collagenous connective tissue fibers and proliferating cells in sensitised and challenged mice as compared to the OVA/OVA-PBS group. Yet, the number of airway smooth muscle cells remained unchanged in the different treatment groups. Budesonide treatment was also effective in reducing the number of goblet cells in the lung sections of OVA/OVA-BUD mice as compared to the OVA/OVA-PBS group.

**Table 3:** Overview of the hitstological and Immunohistochemical score

**Group**	**Inflammation (HE)**	**Goblet cells (PAS)**	**Connective tissue (Masson)**	**α-smooth muscle cells (anti-α-smooth muscle actin)**	**Proliferating cells (anti-Ki67)**
OVA/Sal-PBS	**-**	**-**	**-**	**+**	**-**
OVA/OVA-PBS	**++**	**+**	**++**	**++**	**++**
OVA/Sal-*B. breve*	**-**	**-**	**+**	**+**	**+/-**
OVA/OVA-*B. breve*	**+**	**+**	**+**	**++**	**+**
OVA/Sal-*L. rhamnosus*	**-**	**-**	**+**	**+**	**+/-**
OVA/OVA-*L. rhamnosus*	**+**	**+**	**+**	**++**	**+/-**
OVA/Sal-budesonide	**-**	**-**	**+**	**+**	**+/-**
OVA/OVA-budesonide	**+**	**-**	**+**	**++**	**+**

Slides were reviewed in blinded fashion by two observers independently and slides were scored on the basis of the percentage of positive stained cells in the following way: -,no positive staining; +/-, less than 25% of cells stained positive; +,25 to 50% cells stained positive; ++, 50 to 75% cells stained positive. Results are presented as average score of 5 animals/group.

## Discussion

The aim of this study was to investigate the therapeutic effects of two different beneficial bacterial strains (*B. breve* M-16 V and *L. rhamnosus* NutRes1*)* and a reference treatment (budesonide) on features of the inflammatory response and airway remodelling in a murine model for chronic asthma. The lung resistance was significantly increased in OVA/OVA-PBS mice at all concentrations of methacholine as compared to the OVA/Sal-PBS group. Additionally, the total inflammatory cell number and individual BAL fluid cell counts, except lymphocytes, were significantly increased in OVA/OVA-PBS mice. Increased inflammation score and number of goblet cells, collagenous connective tissue fibers, airway smooth muscle cells and proliferating cells was observed in lung sections of these mice. Hence, our model mimics the airway inflammation and airway remodelling in chronic asthma [2,48,49]. Treatment with *L. rhamnosus or B. breve,* hardly influenced the pathophysiological parameters in OVA/Sal-PBS animals. Interestingly, *L. rhamnosus* and budesonide treatments were effective in suppressing the lung resistance in OVA/OVA mice, significantly decreasing the AHR to methacholine as compared to the OVA/OVA-PBS group. *B. breve* treatment did not affect the AHR to methacholine. Airway inflammation is a characteristic feature of allergic asthma; however, the degree of airway inflammation is not always correlated with the degree of AHR [50]. In the present study, we demonstrate that *L. rhamnosus* prevents the ovalbumin-induced increase in basal airway resistance and AHR at lower concentrations of methacholine. In contrast to *L. rhamnosus*, *B. breve* was not able to significantly reduce changes in airway resistance. Although all the treatments were able to significantly reduce airway inflammation by 50%, there was no complete reduction up till control levels as shown in the BAL fluid as well as in the histological evaluations. These remaining cells might be responsible for the AHR at higher concentrations of methacholine. There is no clear explanation why *L. rhamnosus* did suppress the increase in basal airway resistance and *B. breve* did not. Shifts in cell type such as the reduction in the relative number of macrophages or suppression of specific cytokines such as IFN-γ might explain these observations. The above described results are in accordance with other studies in which beneficial bacteria and budesonide have been individually investigated [1,13,23,24,51-53]. Additionally, *B. breve*, *L. rhamnosus* and budesonide treatments reduced the inflammation score and decreased the number of collagenous connective tissue fibers and proliferating cells in ovalbumin-sensitised and challenged mice as compared to the OVA/OVA-PBS group. Budesonide treatment, but not *B. breve* or *L. rhamnosus,* was also effective in reducing the number of goblet cells in the lung sections of OVA/OVA-budesonide mice. The number of airway smooth muscle cells remained unchanged in the different treatment groups as compared to OVA/OVA-mice.

To our knowledge, we demonstrate here for the first time that *L. rhamnosus* NutRes1 is as effective as budesonide in suppressing lung resistance in chronic allergic mice. Additionally, we demonstrate here that both beneficial bacterial strains are as effective as budesonide in reducing chronic allergic inflammation by attenuating the total inflammatory cell number as well as individual BAL fluid cell counts; and airway remodelling.

PRRs are key components of the innate immunity which are also involved in the activation and shaping of adaptive immunity. The function and expression of PRRs was linked to susceptibility towards allergic asthma. The mRNA expression of *Tlr3* and *Nod1* was significantly decreased in the lungs of OVA/OVA-PBS mice. Previous *in vitro* studies have demonstrated that upon activation by its natural or endogenous ligands, *Tlr3* induces up-regulation of other TLRs, various cytokines and chemokines as well as its own expression and thereby contributes to exacerbation of inflammation [40]. *Nod1* is an intracellular sensor of pathogenic bacteria. Single nucleotide polymorphisms in *Nod1* gene were positively associated with susceptibility towards asthma in farming children and this PRR was reported to be necessary for neutrophil function in mice [25,33-35]. However, no direct associations between *Tlr3* and *Nod1* expression and function and asthma have been reported yet, and whether the decreased mRNA expression of *Tlr3* and *Nod1* caused by the chronic inflammatory status of the animals is pro-inflammatory or anti-inflammatory is also unknown. Moreover, *B. breve* treatment significantly increased *Tlr9* expression. Since TLR9 was shown to exert its immunomodulatory effects on asthma by skewing the increased Th2/Th1 balance towards Th1, this may very well be the situation [37-39]. Budesonide treatment significantly increased the expression of *Tlr3* and *Nod1,* restoring them to normal levels, and raised *Tlr9* (P > 0.05) expression. This further supports a positive role for *Tlr9* and suggests that lowered *Tlr3* and *Nod1* measured in the asthmatic mice is pro-inflammatory in nature [25,34,40]. *L. rhamnosus*, besides significantly increasing *Tlr3* expression and increasing the expression of *Tlr9* expression (P > 0.05), significantly decreased the expression of *Tlr4*. Asthma patients have been shown to have low expression of *TLR4* on their monocytes, lymphocytes and DCs suggesting that this reduction in TLR4 activation might contribute to the disease by reducing the release of Th1 and anti-inflammatory cytokines [54]. These findings might suggest that *L. rhamnosus* treatment in asthmatic mice could not restore the expression of *Tlr4* to its normal level. Taken together, we demonstrate here that the mRNA expression of PRRs in mouse lung tissue is differentially regulated by the different treatments. Yet, all treatments, especially *B. breve*, increased the expression of *Tlr9*.

Th2 cells play a key role in the pathogenesis of allergic asthma, and asthmatic patients were reported to have Th1/Th2 imbalances as well as disturbed Th17/Treg balances. Th2 dominance was observed in the OVA/OVA-PBS group represented by a significant decrease in Th1 and Treg transcription factors and high *Gata3*/*Tbet* ratio. Hence, our model mimics the Th2-responses found in chronic asthma [1,2]. Importantly, *B. breve* shifted the immune balance towards Th1 and Treg, with significantly increased *Foxp3*/*Rorγt* and *Foxp3*/*Gata3* ratios and a significantly decreased *Gata3*/*Tbet* ratio. A different strain of *Bifidobacterium*, *B. animalis*, has been already shown to skew the Th1/Th2 balance towards Th1 in a preventative, acute mouse model for respiratory allergy [22]. Budesonide showed only a moderate effect and significantly increased the expression of *Foxp3,* but did not affect the *Gata3/Tbet, Foxp3/Rorγt, Foxp3/Gata3* and *Foxp3/Tbet* ratios. These findings are consistent with results of other studies which have shown that GCs treatment of asthmatic subjects encourages regulatory responses [55]. *L. rhamnosus* did not influence the mRNA expression of the different Th-specific transcription factors, but significantly decreased the *Gata3*/*Tbet* ratio. More importantly*,* the above observed effects of beneficial bacteria and budesonide on the Th responses in the lung are mirrored by the detection of high Foxp3-expressing Tregs in the blood of treated animals. Tregs play a key role in balancing immune responses and it was demonstrated that an increased expression of Foxp3 in Tregs is directly associated with increased function of these cells [42,56]. No significant differences in the percentage of CD4+ cells that are Foxp3+ were observed. A reduction in the populations of recently activated regulatory T (CD4 + Foxp3 + CD25hi) cells was observed in the treated mice. One possible reason for the loss of these cells may pertain to a reduction in inflammation caused by the treatments. Previous studies have demonstrated that Tregs can become activated through inflammatory signals in the environment which functions as a natural feedback loop by preventing excessive inflammation. An example of this is through TLR2 stimulation [57].

Expression of the pro-inflammatory cytokines *Il1β* and *Il6* was significantly increased in the OVA/OVA-PBS group and expression of both *Il13* and *Il17* was also increased but not significantly. These four cytokines were reported to enhance asthma [30]. *B. breve* treatment, however, significantly increased the expression of the anti-inflammatory cytokine *Il10* and the Th2-associated cytokine *Il4,* yet*,* the mRNA expression of *Gata3* was decreased. Interestingly, the expression of *Tbet* was tightly correlated with *Ifnγ* and *Il12*, while *Foxp3* expression was tightly correlated with *Il10* expression. It has been reported that *IL-12* is involved in the differentiation of Th1 cells and that these cells suppress Th2 cells through the release of *IFN-γ*. Additionally, Tregs suppress other Th cell effector functions through the release of *IL-10*[30]. Budesonide treatment significantly decreased the expression of the pro-inflammatory cytokine *Tnfα* which is in agreement with previously published studies demonstrating the anti-inflammatory effects of budesonide [58]. *L. rhamnosus* treatment significantly decreased the expression of *Il6,* another highly pro-inflammatory cytokine*.* The mRNA expression of Foxp3 was tightly correlated with *Il10* expression*.*

Mast cells are key effector cells in allergic inflammation and mast cell degranulation is detected in asthmatic lungs. The expression of serum mMCP-1 was significantly increased in OVA/OVA-PBS mice as compared to the OVA/Sal-PBS group indicating pulmonary mast cell degranulation. Hence, previous studies demonstrated that mice undergoing a hypersensitivity reaction in the airways have high serum levels of mMCP-1 of pulmonary origin [59]. All treatments were effective in inhibiting mucosal mast cell degranulation, as shown by the significant decrease in the protein levels of mMCP-1 systemically, in serum, in treated mice as compared to the OVA/OVA-PBS group. The mechanism by which these beneficial bacteria reduced mast cells degranulation still needs to be investigated; yet, these effects might be galectines-mediated. Recently, a combination of *Bifidobacterium breve* M-16 V with galacto- and fructo-oligosaccharides was shown to induce galectin-9 release from intestinal epithelial cells which in turn systemically suppressed allergic symptoms, including mast cells degranulation, in mice and humans. The same study demonstrated a negative correlation between increased serum galectin-9 levels and serum mMCP-1 levels [60]. The mechanism by which budesonide affects mast cell degranulation still needs further investigation; yet, glucocorticoids were demonstrated to suppress mast cell activation *in vitro* through the up-regulation of inhibitory regulators of these cells [61]. In addition to their effects on mast cells, *B. breve*, *L. rhamnosus* and budesonide were also effective at reducing cytokine production by T cells in the TLNs. The levels of IL-2, IL-4, IL-6, IL-17a, TNF-α and IFN-γ were significantly increased in anti-CD3-stimulated TLN cells from OVA/OVA-PBS mice as compared to the OVA/Sal-PBS group. IL-2, IL-4, IL-6, IL-17a and TNF-α were reported to be involved in asthma. Increased levels of IFN-γ are found in individuals with severe asthma and acute exacerbation [30,62]. The levels of IL-2, IL-4, IL-6, IL-17a and TNF-α were significantly decreased in OVA/OVA mice treated with *B. breve*, *L. rhamnosus* or budesonide as compared to the OVA/OVA-PBS group. Levels of IFN-γ were significantly decreased in OVA/OVA-*L. rhamnosus* and OVA/OVA-budesonide mice. The level of IL-10 did not differ among the different groups, suggesting that the different treatments could not normalise IL-10 production in chronic allergic mice.

## Conclusions

To our knowledge, this is the first report in which the therapeutic effects of long-term treatment with *B. breve* M-16 V, *L. rhamnosus* NutRes1 and budesonide on asthma are investigated in a murine ovalbumin-induced chronic allergic asthma model. In this current study we show that *B. breve* M-16 V and *L. rhamnosus* NutRes1 are as effective as budesonide in suppressing pulmonary airway inflammation, airway remodelling and inhibiting mast cell degranulation. Additionally, *L. rhamnosus,* but not *B. breve,* reduced lung resistance and IFN-γ production by T cells in the TLNs indicating that treatment effects differ between the bacterial strains. mRNA expression of PRRs, T helper-specific cytokines and transcription factors are differentially modulated by the different treatments. Moreover, *B. breve* induced regulatory responses by increasing *Il10* and *Foxp3* transcription in lung tissue and augmenting the mean fluorescence intensity of Foxp3 in blood CD4+ T cells. These findings show that *B. breve* M-16 V and *L. rhamnosus* NutRes1 may be beneficial in the management of chronic allergic asthma in a therapeutic way.

## Competing interests

Seil Sagar, Mary E Morgan, Si Chen, Johan Garssen, Gert Folkerts and Aletta D Kraneveld are employees of the Utrecht University and declare that they do not have a conflict of interest. Arjan P Vos, Jeroen van Bergenhenegouwen and Niki A Georgiou are employed by Danone Research as indicated in the author affiliations. This aside, there are no personal or financial conflicts of interest to report. Louis Boon is employed by Bioceros B.V. and has no personal financial conflicts of interest to report.

## Authors’ contributions

SS, designed and ran the chronic asthma mouse model, performed all *in vivo* and *in vitro* experiments, collected and analysed data, and wrote the manuscript. MEM, supervised the flow cytometry analysis and advised on data analysis. SC, carried out real-time-PCR analysis, and interpreted and analysed the data. APV and JB provided beneficial bacteria and gave technical support and conceptual advice. ADK, GF, NAG and JG, gave advice on model design, supervised the study and advised on data analysis. LB provided the anti-CD3 antibody. All authors discussed the results and implications and commented on the manuscript at all stages. All authors read and approved the final manuscript.

## Supplementary Material

Additional file 1: Table S1 Primers used for quantitative real-time PCR analysis. Click here for file

Additional file 2: Figure S1 Representative dot plots of T cells analysis in the blood. T cells were isolated on day 56 from whole blood of OVA-sensitised, Sal-challenged (OVA/Sal) mice and OVA-sensitised, OVA-challenged (OVA/OVA) mice treated with PBS (I), *B. breve* (II), *L. rhamnosus* (III) or budesonide (IV; BUD). T cells were gated based on FSC-SSC pattern, followed by analysis of expression of CD4. Then co-expression of Foxp3 and CD25 (regulatory T cells; Treg) was analyzed. Data is representative for n = 6 mice/group. Click here for file
