# Supplementary material for: Bifidobacterium breve and Lactobacillus rhamnosus treatment is as effective as budesonide at reducing inflammation in a murine model for chronic asthma
Source: Respir Res. 2014 Apr 16;15(1):46. doi: 10.1186/1465-9921-15-46 (PMC4029990; doi:10.1186/1465-9921-15-46)
Supplement: Additional file 1: Table S1 — Primers used for quantitative real-time PCR analysis. [file 1465-9921-15-46-S1.docx]

| **Molecule** | **Forward primer** | **Reverse primer** |
| --- | --- | --- |
| ***Tlr1*** | GGTGTTAGGAGATGCTTATGGGG | GATGTTAGACAGTTCCAAACCGA |
| ***Tlr2*** | CCAGACACTGGGGGTAACATC | CGGATCGACTTTAGACTTTGGG |
| ***Tlr3*** | GGGGTCCAACTGGAGAACCT | CCGGGGAGAACTCTTTAAGTGG |
| ***Tlr4*** | GCCTTTCAGGGAATTAAGCTCC | AGATCAACCGATGGACGTGTAA |
| ***Tlr5*** | TCAGACGGCAGGATAGCCTT | AATGGTCAAGTTAGCATACTGGG |
| ***Tlr6*** | GACTCTCCCACAACAGGATACG | TCAGGTTGCCAAATTCCTTACAC |
| ***Tlr9*** | ACTCCGACTTCGTCCACCT | GGCTCAATGGTCATGTGGCA |
| ***Nod1*** | GAAGGCACCCCATTGGGTT | AATCTCTGCATCTTCGGCTGA |
| ***Nod2*** | CCGCTTTCTACTTGGCTGTC | GTGATTTGCAGGTTGTGTGG |
| ***Tbet*** | GCCAGCCAAACAGAGAAGAC | AAATGTGCACCCTTCAAACC |
| ***Gata3*** | GCGGTACCTGTCTTTTTCGT | CACACAGGGGCTAACAGTCA |
| ***Foxp3*** | CACTGGGCTTCTGGGTATGT | AGACAGGCCAGGGGATAGTT |
| ***Rorγt*** | TGCAAGACTCATCGACAAGG | AGGGGATTCAACATCAGTGC |
| ***RPS13*** | GTCCGAAAGCACCTTGAGAG | AGCAGAGGCTGTGGATGACT |

***Supplementary Table 1:*** *Primers used for quantitative real-time PCR analysis*
