# Supplementary figures and images for: Bifidobacterium breve and Lactobacillus rhamnosus treatment is as effective as budesonide at reducing inflammation in a murine model for chronic asthma
Source: Respir Res. 2014 Apr 16;15(1):46. doi: 10.1186/1465-9921-15-46 (PMC4029990; doi:10.1186/1465-9921-15-46)

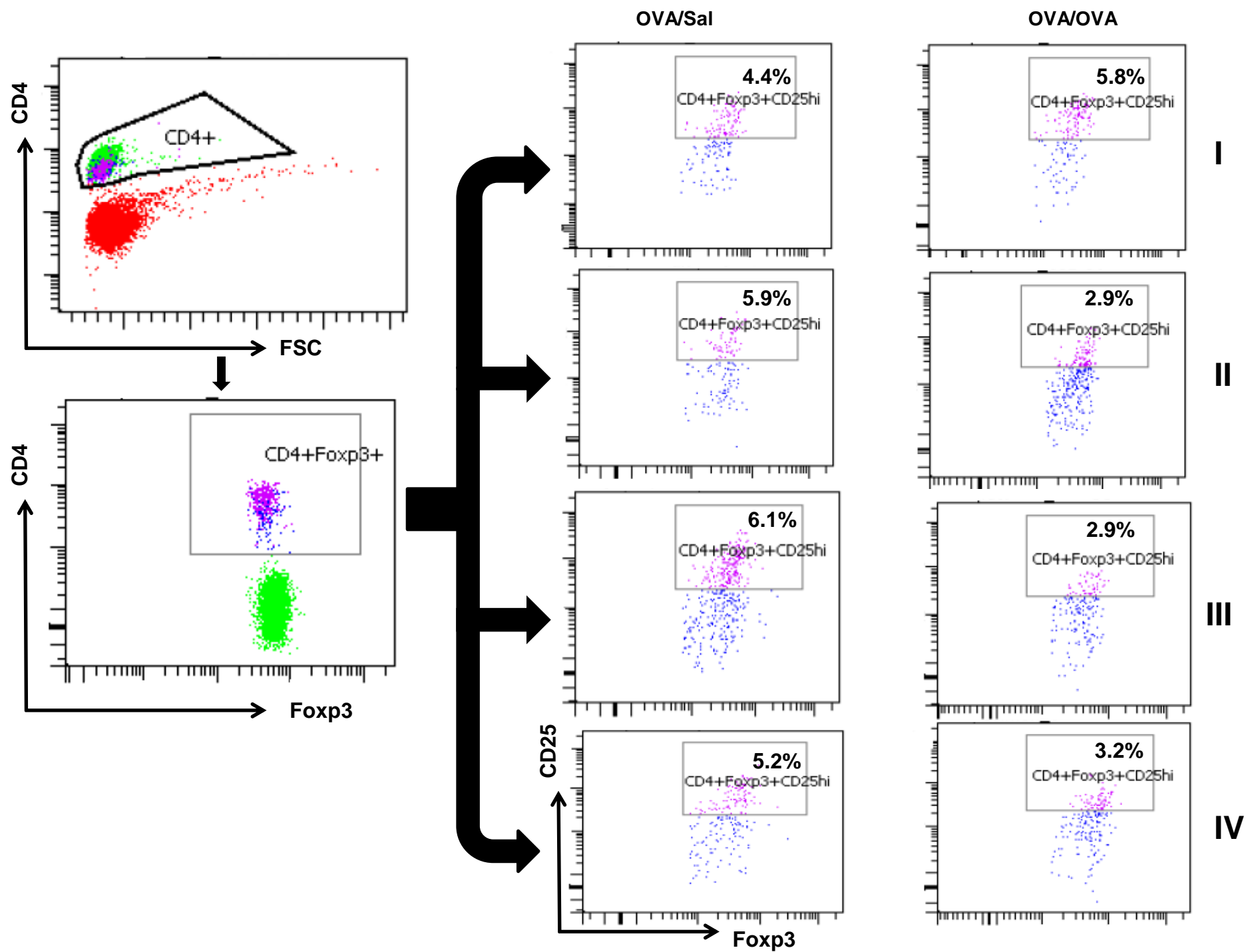

Supplement: Additional file 2: Figure S1 — Representative dot plots of T cells analysis in the blood. T cells were isolated on day 56 from whole blood of OVA-sensitised, Sal-challenged (OVA/Sal) mice and OVA-sensitised, OVA-challenged (OVA/OVA) mice treated with PBS (I), B. breve (II), L. rhamnosus (III) or budesonide (IV; BUD). T cells were gated based on FSC-SSC pattern, followed by analysis of expression of CD4. Then co-expression of Foxp3 and CD25 (regulatory T cells; Treg) was analyzed. Data is representative for n = 6 mice/group. [file 1465-9921-15-46-S2.pdf]
